# Hybrid Immunity for COVID-19 in Bolivian Healthcare Workers

**DOI:** 10.7759/cureus.27449

**Published:** 2022-07-29

**Authors:** Raul Copana Olmos, Nelva Guillen Rocha, Yercin Mamani, Gladys Rodriguez Alvarez, Angelica Ovando Campos, Carla Camacho Tufiño

**Affiliations:** 1 Pediatrics, Universidad Mayor de San Simon, Medicine Faculty, Cochabamba, BOL; 2 Immunology, Hospital del Niño Manuel Ascencio Villarroel, Cochabamba, BOL; 3 Epidemiology and Public Health, Universidad Mayor de San Simon, Medicine Faculty, Cochabamba, BOL

**Keywords:** hybrid immunity, bolivia, hospital, healthcare worker, vaccine, covid-19, sars-cov-2

## Abstract

Introduction

Vaccination is one of the pillars for the prevention of COVID-19 in healthcare workers (HCWs). The present study aims to determine the effectiveness of vaccination for COVID-19 as well as hybrid immunity in previously infected HCWs in a hospital in a developing country.

Methods

An observational study was carried out on health personnel with a complete COVID-19 vaccination schedule according to their previous infection status, with a follow-up period of 15 months.

Results

In this study, 335 subjects were enrolled, of which 32.8% had a previous infection with COVID-19. The safety of vaccines was determined by estimating the presence of adverse effects of vaccination and immunization (AEVI), with the first and second doses showing an incidence of 8.2% and 9.5% respectively, during the second and third waves. Around 5.7% of immunized personnel were sick and 8.4% in the fourth wave; the serum value of neutralizing antibodies was normal at 60.2% with no differences between vaccines (p=0.164). However, in personnel with hybrid immunity, there were normal levels of antibodies in 81.8% of cases (p= 0.023), fewer days of medical leave (6.4 days (standard deviation=1.4) (p=0.067)), higher immunoglobin values ​​(p=0.011) and an insignificantly (p=0.248) lower rate of COVID-19 presentation.

Conclusion

Vaccination, when applied to people who previously acquired natural immunity, generates a hybrid immunity that is robust, and could have a longer duration, as well as greater efficacy for new COVID-19 variants of concern.

## Introduction

In December 2019, an epidemic outbreak of a new severe acute respiratory syndrome (SARS) occurred in Wuhan China. A new coronavirus was identified as the causal agent, initially called nCoV-19 [1]. The World Health Organization (WHO), in January 2020 declared an international health alert, and currently, around 256 million cases and over 5.2 million deaths have been reported worldwide [2]. SARS is a severe form of COVID-19 caused by extensive alveolar damage and progressive respiratory failure [3], pathophysiological mediated by direct injury and inflammation, after the virus entry through spike protein into type II alveolar cells (AT2) of the lung and intestine, as part of the angiotensin-converting enzyme 2 (ACE2) receptor [4]. Knowledge of this mechanism has allowed the development of effective and safe vaccines; Europe started mass vaccination in December 2020, and Asia, America, and Africa started in February 2021; Bolivia received the first doses of Gam-COVID-VAC (Sputnik V) vaccines in January 2021 and BBIBP-CorV (Sinopharm) vaccines in February, and subsequently, immunization of healthcare workers (HCWs) began [5-8]. Natural immunity and vaccine-induced immunity have different mechanisms [9] and certain benefits of vaccinating a previously infected population are reported, known as hybrid immunity; however, it is unknown whether the type of vaccine may have any influence on it, for example, inactivated virus versus mRNA vector vaccines. The present study's aim is to determine the effectiveness of vaccination for COVID-19 as well as hybrid immunity in previously infected HCWs.

## Materials and methods

Study design

We performed an observational study in a COVID-19 referral hospital. HCWs with a complete vaccination schedule (two doses) of both Sinopharm and Sputnik V were included and followed up for 15 subsequent months (from February 1, 2021 to June 1, 2022); patients with pathologies of the immune system or those who received immunosuppressants or immunomodulatory drugs, vaccinated outside the study period or with incomplete doses, or other vaccination schedules were excluded. There was a temporal difference between vaccines since those vaccinated with Sputnik received their first dose in February while those who received the Sinopharm vaccine in March; there was no intervention in the decisions of who vaccinate first or which vaccine to use, so we only referred to the description of what was observed.

Subjects

A total of 335 HCWs were included. A detailed physical examination was carried out on all subjects at the time of administration of the vaccines; an in-hospital follow-up for 30 minutes was performed on all subjects as well after receiving both the first and second dose of the vaccine. Subsequently, follow-up was carried out through telemedicine, and a Google Docs form was used for the notification of adverse reactions according to the schedule for 72 hours at scheduled times; first hour, second hour, sixth hour, noon, first day, second day and third-day post-vaccination.

Neutralizing antibody determination procedure

During a follow-up period of three, six, and 12-15 months, a total of 88 subjects were randomly selected for the determination of neutralizing antibodies. Subjects were informed about the requirements for the sample collection (fasting, lipid-restricted diet for 18 hours, no use of immunomodulatory or immunosuppressive drugs recently), in the adequately prepared patients 5 mL of venous blood was taken, IgG and IgM specific anti-S-RBD levels were performed by chemiluminescence and the study of the neutralizing capacity of antibodies by surrogate antibody neutralization test (sVNT). An informed consent form was signed at the time of sample collection and the results were subsequently returned to each subject by the LABIMED Department of the Faculty of Medicine, San Simon University.

Data processing and statistics

Descriptive statistics were used to describe adverse reactions and effectiveness, Fisher's exact test and Mann-Whitney U test were used for normal samples statistics differences, and a Kaplan-Meier survival test was performed to evaluate differences in COVID-19 presentation between groups

Ethical aspects

The approval of the bioethics committee of the HNMAV was obtained, both the identity and the information collected were confidential, and informed consent was obtained both for the sampling (at the time of sampling) and for the follow-up (at the time of vaccination).

## Results

A total of 335 participants were enrolled in the study. The characteristics of the immunized subjects show a population of HCW with a mean age of 42.8 years, a higher proportion of women to men (3:1), and occupations with 41.6% doctors, 42.8% nurses, and 15.6% other personnel; we observed that older staff (p=0.004), a higher proportion of men (p=0.020) and medical staff (p<0.001) were immunized with Sputnik (see Table 1). Around 32.8% of the staff (n=110) were previously infected by COVID-19 before the first dose of the vaccine (hybrid immunity); these previous infections occurred more than three months prior to the vaccine in 55.5%, between one to three months in 31.8% and in 12.7% less than a month before their first dose; the severity of the infection was 29.1% asymptomatic, 45.5% mild, and in 2.7% moderate to severe. Between the first dose and the second dose, no new cases of COVID-19 infection were reported (see Table 1).

**Table 1:** Baseline characteristics of the vaccinated population groups (n=335). SD: standard deviation

Physical examination at the time of vaccination		Total vaccinated	Sputnik V	Sinopharm	Test
		Media (SD)	Media (SD)	Media (SD)	
Age in years		42.4 (12.9)	44.5 (12.13)	40.8 (13.4)	p=0.004*
Heart rate per minute		79.1 (10.2)	79.7 (10.5)	78.7 (9.9)	p=0.649*
Respiratory rate per minute		20.3 (2.1)	20.7 (2.53)	19.9 (1.47)	p<0.001*
Temperature in C°		36.3 (0.42)	36.2 (0.34)	36.4 (0.47)	p=0.140
Systolic blood pressure in mmHg		114 (13.7)	114.5 (13.6)	112 (14.1)	p=0.550*
Diastolic blood pressure in mmHg		76 (10.13)	76 (10.2)	71 (10.03)	p=0.697*
Healthcare workers characteristics		Count (%)	Count (%)	Count (%)	Test
Sex	Male	81 (24.2%)	45 (30.6%)	36 (19.1%)	p=0.020**
	Female	254 (75.8%)	102 (69.4%)	152 (80.9%)	
Subject***	Physician	133 (41.6%)	75 (55.6%)	58 (31.4%)	p=<0.001**
	Nurse	137 (42.8%)	52 (38.5)	85 (45.9%)	
	Technical staff	39 (12.2%)	7 (5.2%)	32 (17.3%)	
	Administrative staff	11 (3.4%)	1 (0.7%)	10 (5.4%)	
Section of hospital ***	PICU and COVID-19	28 (8.8%)	23 (17%)	5 (2.7%)	p<0.001**
	Emergency and outpatients	25 (7.8%)	16 (11.9%)	9 (4.9%)	
	Hospitalization service	159 (49.7%)	46 (34.1%)	113 (61.1%)	
	Surgery service	69 (21.6%)	43 (31.9%)	26 (14.1%)	
	Complementary services	27 (8.4%)	6 (4.4%)	21 (11.4%)	
	Administrative services	12 (3.8%)	1 (0.7%)	11 (5.9%)	
Previous COVID-19 infectious before vaccine ****		110 (32.8%)	40 (27.2%)	70 (37.2%)	p=0.061**
Severity of the previous infection	Asymptomatic	32 (29.1%)	11	21	p=0.904**
	Mild	50 (45.5%)	20	30	
	Moderate	25 (22.7%)	8	17	
	Severe	3 (2.7%)	1	2	
Time lapsed from the previous infection	<1 month	14 (12.7%)	2	12	p=0.055**
	1 to 3 months	35 (31.8%)	10	25	
	>3 months	61 (55.5%)	28	33	
Transfused blood products		2 (0.6%)	1	1	p=1.000**
* Mann Whitney U test ** Fisher exact test *** 15 lost data **** 33 lost data					

Regarding the safety of vaccines, the presence of adverse effects of vaccination and immunization (AEVI) with the first dose shows an incidence of 8.2% cases, 12.2% of those vaccinated with Sputnik V and 5.3% with Sinopharm (P=0.036), without significant differences in previously infected personnel (p=0.395); During the 72-hour follow-up, it was evident that all these reactions were mild. With the second dose, 9.5% AEVI was reported, 1.5% with Sputnik V and 8.8% with Sinopharm (p=0.005), with no significant differences in previously infected people (p=0.117); all identified reports were of mild local symptoms. We observed that subjects experienced changes during the first 30 minutes of observation in rooms in 9.8% and 2.5%, largely explained by anxiety and fear since no other reactions or immediate adverse events were observed (see Table 2).

**Table 2:** Presentation of adverse effects in the vaccinated population (n=335). SD: standard deviation.

Safety analysis of vaccination		Total vaccinated	Sputnik V ®	Sinopharm ®	Test
		Media (SD)	Media (SD)	Media (SD)	
Presence of AEVI with the first dose**		26 (8.2%)	16 (12.2%)	10 (5.3%)	p=0.036*
Changes in vital signs 30 minutes after the first dose		33 (9.8%)	24 (16.3%)	9 (4.8%)	p=0.001*
Mild or local symptoms reported during 72 hours following the first dose	None	295 (88%)	122 (83%)	173 (92%)	p=0.066*
	Pain at the puncture site	27 (8.1%)	17 (11.6%)	10 (5.3%)	
	Headache	15 (4.5%)	5 (3.4%)	10 (5.3%)	
	Fever	3 (0.9%)	1 (0.7%)	2 (1%)	
	Drowsiness	3 (0.9%)	2 (1.4%)	1 (0.5%)	
Presence of AEVI with the second dose**		30 (9.5%)	2 (1.5%)	28 (8.8%)	p=0.005*
Changes in vital signs 30 minutes after the second dose		8 (2.5%)	7 (5.5%)	1 (0.5%)	p=0.009*
Mild or local symptoms reported during the first 72 hours following the first dose	None	274	118 (92.8%)	156 (84.8%)	p=0.095*
	Headache	6 (1.9%)	1 (0.8%)	5 (2.7%)	
	Pain at the puncture site	31 (9.9%)	8 (6.3%)	23 (12.5%)	
Moderate or severe symptoms during the 72 hours after the first and second dose		0 (0%)	0 (0%)	0 (0%)	p=1.000*
* Fisher exact test ** 16 patients were lost due to not completing at least one of seven follow-ups (seven cases in first dose and 24 in the second dose follow-ups)					

**Figure 1:**
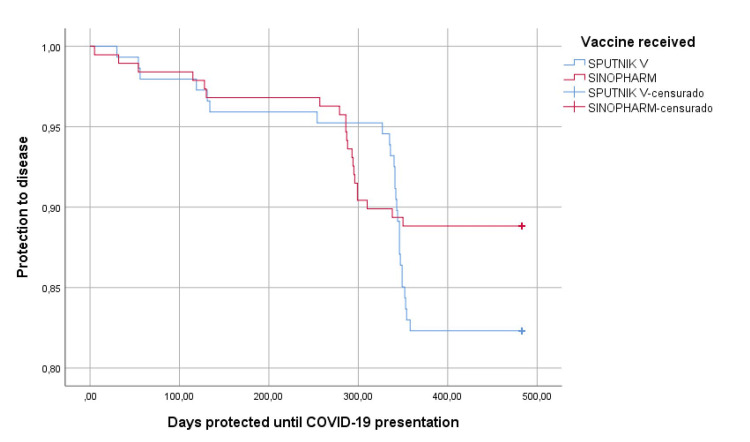
Protection from disease according to the type of vaccine received (n=335). censurado: censored cases

Regarding the effectiveness and protection against COVID-19 infection during the second and third waves, we found that 5.7% (19) people presented symptoms of COVID-19 infection, of which 5.4% of those vaccinated with Sputnik V and 5.9% with Sinopharm (p=0.872), higher rate during the fourth wave since 8.4% (28) people got infected, 12.2% of those immunized with Sputnik V and 5.3% with Sinopharm (p=0.028); in general, during whole follow-up period protection from the disease showed no differences between vaccines (p = 0.117) (Figure 1). The severity of the infection in 47 vaccinated people who got infected was: 14.9% were asymptomatic, 80.9% had mild symptoms, and 4.3% had moderate to severe infections, with no differences between the type of vaccines (p=0.088) (Table 3). The value of neutralizing antibodies was normal in 60.2% with no differences between vaccines (p=0.164), but higher IgG values in those vaccinated with Sputnik (p=0.028); The average number of days of illness was 9.6 (S.D.5.4) higher in those vaccinated by Sinopharm (p=0.038).

**Table 3:** Clinical efficacy and humoral response to immunizations (n=335). SD: standard deviation.

Efficacy analysis of vaccination		Total vaccinated	Sputnik V ®	Sinopharm ®	Test
		Count (%) / Mean (SD)	Count (%) / Mean (SD)	Count (%) / Mean (SD)	
New COVID-19 infection during 2nd and 3rd wave		19 (5.7%)	8 (5.4%)	11 (5.9%)	p=0.872*
New COVID-19 infection during 4th wave		28 (8.4%)	18 (12.2%)	10 (5.3%)	p=0.028*
Infectious severity symptoms (n=47)	Asymptomatic	7 (14.9%)	6 (30%)	1 (4.8%)	p=0.088*
	Mild	38 (80.9%)	20 (70%)	18 (85.8%)	
	Moderate	2 (4.3%)	0 (0%)	2 (9.6%)	
	Severe	0 (0%)	0 (0%)	0 (0%)	
Neutralizing antibodies level***	Normal	53 (60.2%)	46 (63.9%)	7 (43.8%)	p=0.164*
	Low	35 (39.8%)	26 (36.1%)	9 (56.3%)	
Days of vaccine protection until COVID-19 infection		173 (121.9)	161.6 (119.7)	182.4 (128.8)	p=0.881**
Day of illness		9.6 (5.4)	6.8 (3.76)	11.9 (5.54)	p=0.038**
IgG anti-RBD COVID-19 serum values		0.27 (0.40)	0.2 (0.4)	0.4 (0.42)	p=0.028**
IgM anti-RBD COVID-19 serum values		1.8 (5.2)	0.9 (1.58)	4.1 (9.39)	p=0.679**
* Fisher exact test ** Mann Whitney U test *** 88 neutralizing antibodies serum test performed					

**Figure 2:**
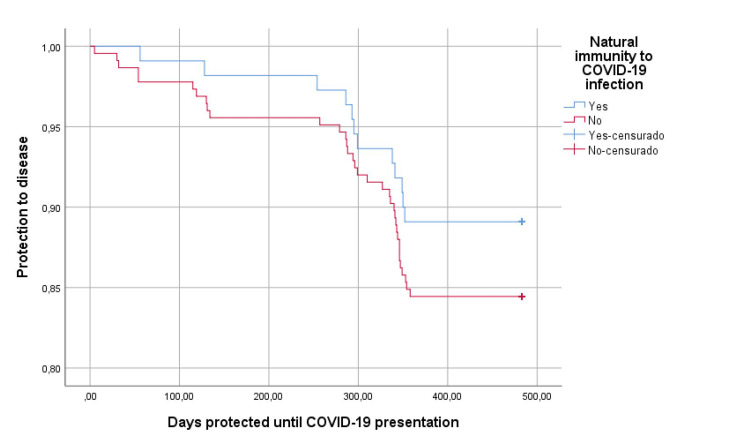
Protection against COVID-19 disease according to hybrid immunity censurado: censored cases

**Figure 3:**
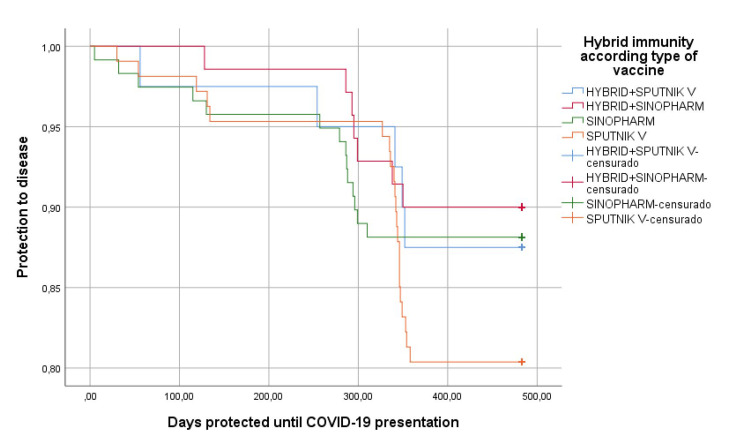
Protection against COVID-19 disease according to hybrid immunity and type of vaccine received censurado: censored cases

In relation to the protective effect of hybrid immunity, we observed normal antibody levels in 81.8% of cases (p=0.023), which was much higher than in vaccine-induced immunity alone, and had fewer days of illness to 6.4 days (D.S.1.4) (p=0.067), higher IgM values ​​(p=0.011) (Table 4); in the same way, slight differences (p=0.248) were found regarding protection from disease in the personnel with hybrid immunity or according to those who were vaccinated with inactivated agents than with mRNA vectors (p=0.286) (Figures 2-3).

**Table 4:** Clinical efficacy and humoral response associated with hybrid immunity (n=335). SD: standard deviation

		Hybrid immunity (n=110)	Vaccine-induced immunity(n=225)	Test
		Count (%) / Mean (SD)	Count (%) / Mean (SD)	
New COVID-19 infection during 2nd and 3rd wave		5 (4.5%)	14 (6.2%)	p=0.623*
New COVID-19 infection during 4th wave		7 (6.4%)	21 (9.3%)	p=0.346*
Infectious severity symptoms (n=47)	Asymptomatic	2 (16.7%)	5 (14.3%)	p=1.000*
	Mild	10 (83.7%)	28 (80%)	
	Moderate	0 (0%)	2 (5.7%)	
	Severe	0 (0%)	0 (0%)	
Neutralizing antibodies level***	Normal	18 (81.8%)	35 (53%)	p=0.023*
	Low	4 (18.2%)	31 (47%)	
Days of vaccine protection until COVID-19 infection		278 (93.7)	254 (117.24)	p=0.526**
Day of illness		6.42 (1.38)	9.03 (4.71)	p=0.067**
IgG anti-RBD COVID019 serum values		0.36 (0.53)	0.24 (4.71)	p=0.383**
IgM anti-RBD COVID-19 serum values		5.02 (9.05)	0.61 (1.48)	p=0.011**
Neutralization of antibodies percentage		69.7 (40)	36.7 (42.4)	p=0.002**
* Fisher exact test ** Mann Whitney U test *** 88 neutralizing antibodies serum test performed				

## Discussion

The HCWs of the tertiary care hospital studied are young, predominantly female, with a balanced distribution of medical, nursing, and administrative personnel; the first vaccine available in February 2021 (Sputnik V) was administered on a priority basis to older personnel, doctors, and nurses in critical areas; the second vaccine (Sinopharm) applied in March was aimed at younger staff, nurses, administrative staff from the remaining clinical areas. This reflects the difficulty in making ethical decisions when prioritizing the administration of a vaccine in HCW [10] in contexts of scarcity, thus establishing some real facts: priority of immunization of doctors versus nurses; men versus women, for certain services over others. One explanation for this phenomenon is that in the first phase of vaccination in Bolivia, the Ministry of Health prioritized medical personnel, those aged 60 years and above, and people with underlying diseases; once this phase was completed those between 18 and 59 years old became eligible for vaccination throughout the country, except pregnant women and children. However, the decision of which HCW to vaccinate first raises ethical questions about the principles applied at the time of selection; anxiety and physiological responses of fear were observed in the personnel at the time of vaccination. This point allows us to visualize that in future health crises and in contexts of scarce resources, more detailed ethical guidelines should be worked out to allow us to take a more appropriate decision [11].

The Sputnik V vaccine (Gam-COVID-Vac) has support and consistent evidence [12], uses a recombinant heterologous adenovirus approach (adenovirus 26 and 5), arrived in Bolivia in January 2021, and doses were administered to HCWs in February. In February 2021, the Sinopharm vaccine arrived in Bolivia, produced by the laboratory of the Institute of Biological Products from Beijing - China [13], using the inactivated strains 19nCoV-CDC-Tan-HB02 (HB02), 19nCoV-CDC-Tan-Strain03 (CQ01) and 19nCoV-CDC-Tan-Strain04 (QD01). Our study, which is very similar to clinical trials of vaccines, reflects a low rate of mild adverse effects both in the first and second dose (<10%) similar to studies carried out in Buenos Aires that describe the incidence of AEVI in 700 HCW after immunization with Sputnik V, 57% of participants reported pain at the injection site, 11% redness and swelling, and 5% diarrhea [7]. Another study of 1029 HCWs from Iraq reported that 54.9% of workers vaccinated with Sinopharm presented mild symptoms as previously described [7,8]; our study did not find any other clinical manifestations reported by staff in the long-term vaccinated population.

Immunogenicity and humoral protective effect through the activity of the neutralizing antibodies showed a decrease after six months in about 40% HCW with no difference between vaccines, however, in patients with hybrid immunization reached normal levels of neutralizing antibodies in 80% of the cases, which demonstrates a longer and more effective duration of the immune system and protection for different variants (second, third, and fourth wave); mainly given by the presence of IgM compared to IgG in people with only vaccinated induce immunity [14]; which is consistent with studies describing protection against new strains, including Alpha B.1.1.7 (first identified in the UK), Beta B.1.351 (first identified in South Africa), Gamma P.1 (identified for the first time in Brazil), Delta B.1.617.2 and B.1.617.3 (first identified in India) and the Omicron variants B.1.1.529 BA.1 and BA.2 [14-16]. Both vaccines demonstrated an important protective effect on HCWs. There were 110 people who became ill prior to the vaccine, 2.7% had severe symptoms, 22.7% had moderate symptoms, 74.6% had mild or asymptomatic symptoms; while after the vaccine 47 people became ill, with no severe symptoms, 4.2% moderate, 95.8% mild and asymptomatic (p<0.001). It becomes much more significant in patients with hybrid immunity, of whom 100% of the cases were mild or asymptomatic, probably because natural immunity improves the humoral immunity, B lymphocytes response, T lymphocyte CD4+ and CD8 cell immunity to variants, which is powered with immunization generating an effect of "vigorous hybrid immunity" according to similar studies [9,15-17].

Variants of concern have increased transmissibility rates as they greater ability to evade immunity, and have resulted in lower efficacy of first-generation vaccine protection, making it necessary to administer boosters in view of a drop in the effectiveness of the original two-dose scheme [18]. Our study shows that people with hybrid immunity have a longer protection time than those who were only immunized with two doses vaccine scheme [19], and supports the need for boosters to prolong the protective effect of vaccines [20]; these preventive measures could help in reducing mortality from COVID-19, which is very important in countries with limited resources [21-23]. The study has limitations concerning the lack of information about the third and fourth boosters because this information was not included, due to the multiple booster administration schedules used in our country.

## Conclusions

We conclude that vaccination, when applied to people who have acquired natural immunity, generates a hybrid immunity that is robust and improves the effectiveness of vaccination against COVID-19, with some influence on reducing the severity of illness, and with higher humoral response values. However, more studies are required to specify the nature of these differences and their possible uses in other pathologies.
